# Physical Inactivity Among University Students: A Cross-Sectional Study at King Faisal University, Saudi Arabia

**DOI:** 10.7759/cureus.93911

**Published:** 2025-10-06

**Authors:** Hussain A Aldehneen, Mariya E Almohammedali, Zahrah M Almadeh, Fatima K Albari, Rainad A Alruwaili, Atheer S Farhan, Fajer S Alsharif, Zahra A Albashrawi, Maitham N Alsarhan

**Affiliations:** 1 Department of Family Medicine, Almoosa Specialist Hospital, Alahsa, SAU; 2 College of Medicine, Vision Colleges, Riyadh, SAU; 3 Department of Family Medicine, Alahsa Health Cluster, Alahsa, SAU; 4 Department of Respiratory Therapy, Eastern Health Cluster, King Fahad Specialist Hospital, Dammam, SAU; 5 Department of Family Medicine, Family Medicine Academy, Ahsa, SAU

**Keywords:** al-ahsa, kfu, physical activity, saudi arabia, university students

## Abstract

Background: Physical inactivity is considered a leading cause of morbidity and mortality worldwide. Studies indicate that sedentary lifestyle patterns are prevalent among segments of the Saudi population. University students have been reported to show lower physical activity levels associated with academic stressors, increasing their risk of many diseases, particularly obesity.

Aim: This study aims to evaluate the adherence to the World Health Organization (WHO) recommendations of physical activity among King Faisal University (KFU) students and to examine the difference between medical and non-medical college students.

Methods: This cross-sectional survey-based study was conducted in late 2023 using an English version of the Global Physical Activity Questionnaire (GPAQ) and questions about certain sociodemographic factors, such as academic level, age, and some habits, such as smoking, among KFU students. Associations were examined using chi-square/Fisher’s exact tests and multivariable logistic regression.

Results: A total of 558 participants were included in the analysis, with 186 (32.8%) found to be physically inactive. The difference in physical inactivity was significant between participants from medical and non-medical colleges. Being a medical student increased the odds of being physically inactive by 63.5%, and smoking increased the odds by 222.25%.

Conclusions: Physical inactivity was highly prevalent among KFU students and significantly higher among medical students. Beyond its notorious effect on health, smoking also seemed to dictate the behavior and decrease the level of physical activity.

## Introduction

Physical inactivity is considered globally the fourth leading factor of mortality, giving rise to multiple non-communicable diseases (NCDs) such as cardiovascular disease, diabetes mellitus, and cancer, along with their consequences like raised blood pressure, raised blood sugar levels, overweight, and obesity [1]. This inactivity and adherence to a healthy lifestyle tend to decline upon entering college life, which contributes to a high risk of developing cardiovascular disease in the future, especially ischemic heart disease [2,3]. The Global Physical Activity Questionnaire (GPAQ) has been developed by the World Health Organization (WHO) to help in measuring the activity of an individual according to the global guidelines [4]. Global recommendations by WHO encourage performing at least 150 minutes of moderate-intensity aerobic physical activity or at least 75 minutes of vigorous-intensity aerobic physical activity throughout the week for adults aged 18-64 [1].

Studies indicate that sedentary lifestyle patterns are prevalent among segments of the Saudi population in which both genders do not meet the recommended physical activity according to global guidelines [5]. This could contribute to multiple diseases such as coronary artery disease (CAD), obesity, and diabetes mellitus [6-9]. Obesity, on one hand, accounted for more than half of the Saudi population in 2017 and is estimated to exceed 59% by 2022, with higher rates in females in relation to physical inactivity [8,10]. Furthermore, diabetes in Saudi Arabia is rising with higher rates in females, children, and those who live in urban areas [9].

Previous cross-sectional studies in Saudi Arabia have documented high levels of physical inactivity among medical students, highlighting the need for targeted interventions [11]. University students have been reported to show lower physical activity levels associated with academic stressors. [12]. Males have lower physical activity compared to females [3,13]. Among university students, about 19% are considered overweight, while about 6% are obese [14]. Limited data exist on the lifestyle and physical activity of KFU students. Many do not meet recommended activity levels, increasing their risk of noncommunicable diseases. This study aims to assess the prevalence of physical inactivity and its associated risk factors among KFU students.

## Materials and methods

Study design and setting

This quantitative, descriptive, cross-sectional questionnaire-based study was conducted from August 2023 to December 2023 at KFU, Hofuf, Al-Ahsa, Eastern Province, Saudi Arabia. KFU is one of the largest universities in the country, with more than 50,000 students enrolled across 15 colleges, including four healthcare colleges (Medicine, Clinical Pharmacy, Dentistry, and Applied Medical Sciences) and 11 non-healthcare colleges (Agriculture and Food Science, Veterinary Medicine, Education, Business, Science, Computer Science, Engineering, Art, Law, Applied Studies-Hofuf, and Applied Studies-Baqiq).

Study population

The study population included all KFU students residing in Al-Ahsa during the 2023-2024 academic year. Students from other universities, those living outside Al-Ahsa, or not enrolled in 2023-2024, were excluded. A total of 1,500 students were invited to participate in the study, yielding 609 respondents (response rate, 41%), of whom 558 (91.6% of respondents; 37.2% of invited) were eligible for analysis.

Sampling technique

The sample size was calculated using Richard Geiger’s equation with a confidence level of 95%, a margin of error of 5%, and a total population of approximately 50,000 students. To ensure sufficient statistical power and to account for potential missing data, the final required sample size was increased accordingly [15]. A non-probability sampling method was adopted because of the large population size. A convenience sampling technique was applied, whereby the main researcher, with the assistance of four colleagues, distributed a QR code leading to a Google Form of the questionnaire.

Data collection

Data were collected via an online Google form that contained a validated, pretested, self-administered electronic questionnaire. The questionnaire consisted of 23 items divided into two sections. The first section contained seven questions covering socio-demographic characteristics (age, gender, marital status, college, academic level, living situation, and smoking status). The second section included 16 questions adapted from the Global Physical Activity Questionnaire (GPAQ), developed by the World Health Organization (WHO), which assesses physical activity across work, transport, and recreational domains. It distinguishes between vigorous and moderate activities and records the number of days per week and the time spent per day on each activity.

The Metabolic Equivalent of Task (MET) score was calculated according to WHO GPAQ analysis guidelines. MET is defined as the ratio of a person’s working metabolic rate relative to the resting metabolic rate. One MET equals the energy cost of sitting quietly (equivalent to 1 kcal/kg/hour). For analysis, 4 METs were assigned to time spent in moderate activities and 8 METs to time spent in vigorous activities. Permission to use and adapt the GPAQ was not required, as it is a publicly available tool provided by the WHO for research and public health surveillance. Then, the raw data was stored in Excel sheets.

Statistical analysis

Data were analyzed using the Statistical Package for the Social Sciences (SPSS) version 26 (IBM Corp., Armonk, NY). Descriptive statistics (means, standard deviations, frequencies, and percentages) were calculated. The chi-square test of independence and Fisher’s exact test were used to assess associations between sociodemographic factors and meeting the WHO physical activity recommendations. Binary logistic regression was performed to identify predictors of meeting these recommendations. A *P*-value ≤ 0.05 was considered statistically significant for all tests.

## Results

Out of a total of 609 respondents to the questionnaire, 558 (91.6% of respondents) were eligible for analysis. The mean age was 20.9 ± 1.7 years, with the majority aged 20-22 years (342, 61.3%). More than half were female (357, 64.0%), and 354 (63.4%) were from medical colleges. Most students were single (474, 84.9%), and the largest proportion were in their fourth academic year (153, 27.4%). Regarding lifestyle factors, 45 students (8.1%) were smokers, while the remainder were either never-smokers or non-smokers. Detailed sociodemographic characteristics are presented in Table 1.

**Table 1:** Sociodemographic data of the participants (n = 558). Values are *n* (%) unless otherwise indicated; age presented as mean ± SD. SD, standard deviation

Sociodemographic data	n	%
Gender		
Male	201	36.0
Female	357	64.0
Age (years)		
18-19	120	21.5
20-22	342	61.3
23-25	96	17.2
Mean ± SD (age in years)	-	20.9 ± 1.7
Married	84	15.1
Single	474	84.9
College		
Non-medical	204	36.6
Medical	354	63.4
Preparatory year	102	18.3
Second year	78	14.0
Third year	99	17.7
Fourth year	153	27.4
Fifth year	90	16.1
Sixth year	33	5.9
Internship	3	0.5
Living with a roommate (off-campus)	12	2.2
Living alone (off-campus)	18	3.2
Living with family	492	88.2
Student housing (in-campus)	36	6.5
Never-smoker	477	85.5
Non-smoker	36	6.5
Smoker	45	8.1

A chi-square test of independence was performed to examine associations between sociodemographic variables and meeting the WHO physical activity recommendations. Significant associations were observed between age and the level of activity (*P* = 0.007). Students in the 23-25 age group reported the highest rate of inactivity (42, 43.8%), while those aged 20-22 reported the lowest (96, 28.1%). College type was also significantly correlated (*P *= 0.004), with medical students demonstrating a significantly higher prevalence of inactivity (132, 37.3%) compared to their non-medical peers (51, 25.0%). Moreover, a strong association was also found with academic level (*P *< 0.001), where inactivity peaked among fifth-year students (48, 53.3%) and was lowest among third-year students (15.2%). Living status (*P *= 0.002) and smoking status (*P *= 0.008) were also significant. Notably, half of the students in university housing (18/36, 50.0%) were inactive, and smokers were substantially more likely to be inactive (24/45, 53.3%) than those who had never smoked (159/513, 31.4%). On the other hand, no significant associations were found for gender (*P *= 0.302) or marital status (*P *= 0.449). Full details are given in Table 2.

**Table 2:** WHO physical activity requirements in relation to the sociodemographic data (n = 558). Note: The chi-square test of independence was used to assess associations. *A *P*-value ≤ 0.05 was considered statistically significant. Some categories were combined for clarity. WHO, World Health Organization

Variable	Category	Total (*N*)	Number inactive (*n*)	Inactivity rate (%)	*P*-value
Gender	Male	201	60	29.9	0.302
-	Female	357	123	34.5	
Age	18-19	120	45	37.5	0.007*
	20-22	342	96	28.1	-
	23-25	96	42	43.8	-
Marital status	Married	84	24	28.6	0.449
	Single	474	159	33.5	-
College	Non-medical	204	51	25.0	0.004*
	Medical	354	132	37.3	
Academic level	Preparatory year	102	39	38.2	<0.001*
	Second year	78	21	26.9	-
	Third year	99	15	15.2	-
	Fourth year	153	51	33.3	-
	Fifth year	90	48	53.3	-
	Sixth year and Intern	36	9	25.0	-
Living status	With family	492	156	31.7	0.002*
	Student housing	36	18	50.0	-
	Off-campus alone	18	9	50.0	-
	Off-campus w/roommate	12	0	0.0	-
Smoking status	Never/Non-smoker	513	159	31.0	0.008*
-	Smoker	45	24	53.3	-

To further identify independent predictors of meeting WHO recommendations, a binary logistic regression was conducted, including age, college, academic level, living status, and smoking status. The model was significant (χ²(13) = 67.078, *P* < 0.001), with Nagelkerke *R*² = 0.158, indicating a modest effect size. The analysis identified two significant predictors: college type and smoking status. After controlling for other factors, students in medical colleges had 1.64 times the odds of being physically inactive compared to students in non-medical colleges (odds ratio (OR) = 1.64, *P* = 0.024).

Smoking status emerged as the strongest predictor. The odds of being physically inactive were 3.26 times higher for smokers than for non-smokers (OR = 3.26, *P* = 0.006).

In the final model, age, academic level, and living status were not found to be significant predictors of physical inactivity. The full logistic regression results are presented in Table 3.

**Table 3:** Binary logistic regression with predictors of meeting WHO requirements. Binary logistic regression model where the outcome was failing to meet the WHO physical activity recommendations. *B* represents the logistic coefficient, and OR is the odds ratio. An OR > 1 indicates increased odds of being physically inactive. The overall model was statistically significant (χ²(13) = 67.078, *P* < 0.001), and the Nagelkerke *R*² was 0.158. *A *P*-value ≤ 0.05 was considered statistically significant. WHO, World Health Organization

Variable	B	*P*-value	OR
Age	0.132	0.306	1.141
College	0.492	0.024*	1.635
Academic level	-20.235	0.996	1.00
Living status	21.51	0.816	1.00
Smoking status	1.18	0.006*	3.255

**Figure 1:**
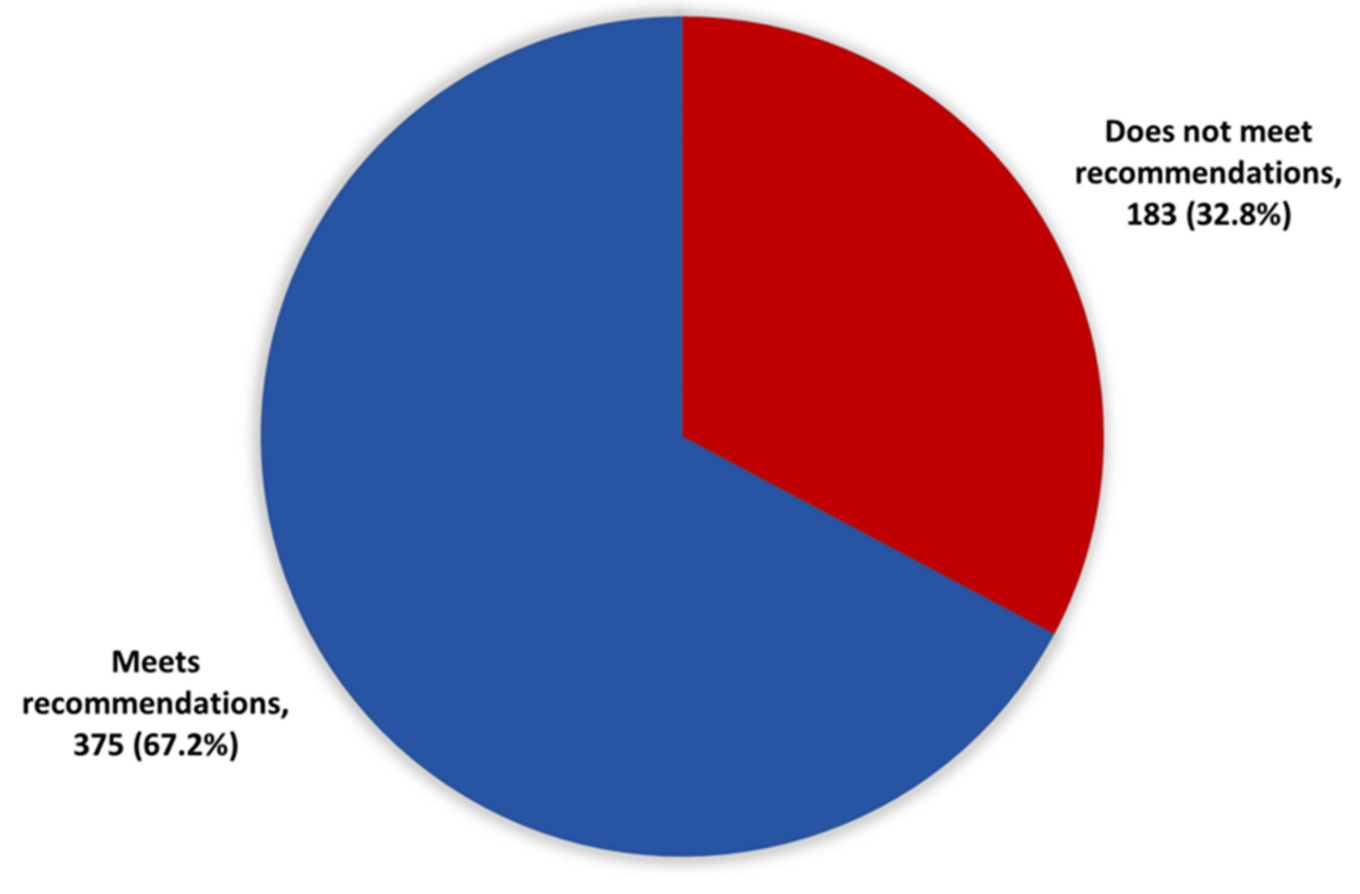
Compliance with WHO physical activity recommendations. WHO, World Health Organization

Proportion of KFU students meeting the WHO physical activity recommendations. Among the 558 participants, 186 participants (32.8%) did not meet the WHO weekly physical activity recommendations, whereas 372 participants (67.2%) successfully met these recommendations, as shown in Figure 1.

**Figure 2:**
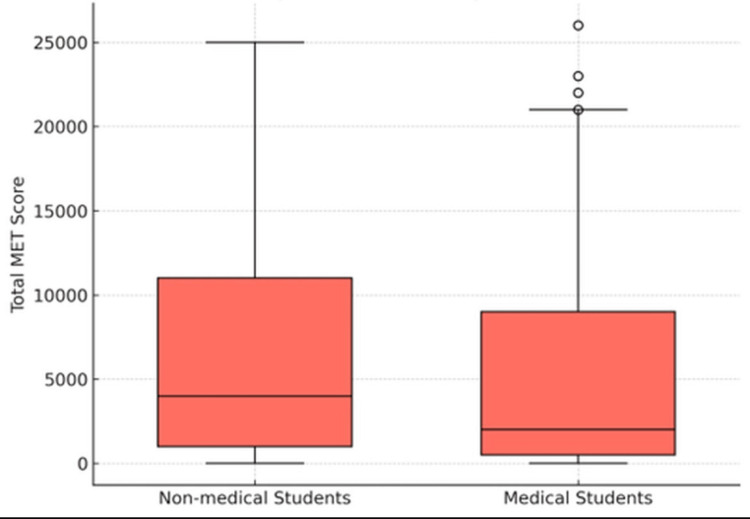
Boxplot of total MET scores comparing KFU students from medical and non-medical colleges. MET, Metabolic Equivalent of Task; KFU, King Faisal University

Figure 2 shows that non-medical students demonstrated a higher median total MET (~4,000) with a broader interquartile range (1,000-11,000) and a higher maximum (~25,000) compared to medical students (median ~2,000; IQR 500-9,000; maximum ~21,000). Several outliers (>21,000) were observed among medical students.

## Discussion

This descriptive cross-sectional study assessed the prevalence of physical inactivity and its associated factors among students at KFU in Al-Ahsa, Saudi Arabia, during 2023-2024. Among the 558 participants, 183 (32.8%) did not meet the WHO weekly physical activity recommendations, while 375 (67.2%) met the recommended levels. From a local perspective, the prevalence of inactivity in our sample (32%) was lower than the 96% reported by Al-Nozha et al. in the general Saudi population [5], likely reflecting differences in study period, methodology, and the specific focus on university students who are in a younger age group. Comparable findings of high inactivity among Saudi medical students were also documented by Khalafalla et al. [11]. These findings are additionally consistent with observations in Canadian and American colleges where students failed to engage in sufficient physical activity [16]. For instance, in Canadian colleges, many students did not achieve a sufficient level of physical activity.

In our study, we found that students in medical colleges had 1.64 times the odds of being physically inactive than students in non-medical colleges. Cross-sectional studies in Saudi Arabia have similarly reported elevated inactivity among health and medical students. At King Khalid University, 47.5% reported inactive leisure time, and multivariable models identified independent predictors of inactivity among health college students, citing time constraints and perceived barriers that map to academic demands [17]. At Al-Jouf University’s College of Medicine, medical students reported marked barriers, including lack of time and academic workload; patterns were consistent with lower engagement in moderate-to-vigorous activity relative to guidelines [18].

Comparable results have been reported in other Asian contexts. Naim et al. reported inactivity rates of 49% versus 35% among medical and non-medical students, respectively [19]. More Asian regions reported similar findings, where regional physical inactivity averages around 44% [20]. The consistently higher rates among medical students may be explained by their demanding academic schedules and prolonged study hours [21], although further longitudinal research is warranted to examine underlying causes and trends over time.

Living arrangements were also significantly associated with physical inactivity. Students residing on campus at KFU showed higher inactivity compared to those living off campus with roommates. To our knowledge, this association has not been examined in previous literature. A plausible explanation is the limited availability of recreational facilities such as gyms and sports areas within KFU housing at the time of data collection, along with the absence of motivational peer support that roommates can provide in off-campus shared accommodations. This finding highlights the importance of both physical and social environments in shaping physical activity behavior. KFU and similar institutions should implement health-promotion programs focused on integrating regular physical activity into academic life. Establishing on-campus recreational facilities, designing awareness campaigns, and combining physical activity promotion with smoking cessation strategies are recommended.

In our study, we found that smoking status was the strongest predictor of inactivity, with smokers having 3.26 times higher odds of being inactive than non-smokers. While fewer Saudi student studies quantify this association with odds ratios, converging evidence links unhealthy lifestyle clustering; students who smoke often report lower physical activity and higher sedentary time, especially during stress periods such as examinations. Broader regional and international literature among adolescents and young adults indicates that older age within the student range and smoking co-occur with lower physical activity, plausibly mediated by social routines, stress coping, and sleep dysregulation. Pandemic-era multicenter surveys in university populations demonstrated that psychological distress, isolation, and altered routines were associated with increased inactivity; within these contexts, smokers were often at elevated risk for reduced activity relative to peers. Mechanistically, nicotine use can be embedded in low-activity social contexts and may co-occur with late sleep timing and sitting, reinforcing sedentary patterns; in addition, perceptions of exertional discomfort may be higher among smokers. Clinically and programmatically, this finding suggests that physical activity promotion and smoking cessation should be integrated within campus health services; combined behavior-change interventions and peer-led programs might leverage social networks to concurrently reduce smoking and increase physical activity, with evaluation using objective activity monitoring [22-25].

In our study, we found that inactivity varied significantly by age and academic level; inactivity peaked in the fifth year and was higher in the 23-25 age group, while third-year students reported the lowest inactivity. Age gradients for physical activity in Saudi youth and young adults have been observed, with increasing age associated with reduced physical activity; among male adolescents in Riyadh, older age was negatively associated with physical activity behavior, reflecting escalating academic demands and fewer opportunities for organized sport. Academic seniority often coincides with intensive coursework, clinical rotations, or capstone requirements, which reduce discretionary time for exercise; similar patterns were noted among health colleges in the southwest, where perceived barriers included study load and fatigue. Pandemic-era disruptions also interacted with academic progression, with students engaged in clinical or lab-based programs reporting complex constraints on activity opportunities. Although campus-specific factors vary, these data collectively support a time-availability and environment hypothesis. Future studies at KFU should longitudinally track cohorts across academic years, measure timetable density, commuting, and step counts, and test schedule-level interventions such as embedded micro-activity breaks and credit-bearing physical activity electives in senior years [18,22,24-25].

In our study, we found that gender and marital status were not significantly associated with meeting WHO physical activity recommendations, despite higher inactivity commonly reported among female students in Saudi settings. Several Saudi university studies have reported high overall inactivity prevalence and, in many cases, higher inactivity among women; for example, a King Saud University survey reported 62.5% inactivity with a large proportion of female respondents, and a mixed-methods national study found that 70% of female university participants did not meet WHO recommendations, citing limited facilities and social constraints. Other university samples, including Umm Al-Qura University, reported that 54% of students failed to meet recommendations overall and did not always observe large gender differences in light-intensity activity, suggesting heterogeneity by campus and measurement. The lack of a significant gender difference in the present study may reflect the university's specific facility provisioning, sample composition with a majority of medical students, or residual confounding by academic level and living status. It could also reflect increased access to women's sports facilities in recent years. Future research should incorporate objective measures such as accelerometry, stratify by facility access and time of day, and include qualitative inquiry to parse gendered barriers that may not fully manifest in aggregate statistical comparisons [26-27].

The timing of this study during a global trend of digital solutions and an intense online presence may also explain the relatively high prevalence of inactivity. Moreover, global evidence has demonstrated reductions in physical activity during and after pandemic-related lockdowns, and such effects may persist.

Strengths and limitations

The study benefited from multiple factors, including the use of a validated and pretested questionnaire and a large, diverse sample representing multiple colleges within KFU. Despite the fact that we did our best and dedicated all available resources, a cross-sectional study design cannot establish causality. So, a future prospective study is needed to establish causality as well as to eliminate the possible confounding effect of the timing and season of the year. Moreover, despite distributing the questionnaire across multiple colleges and through multiple data collectors, a convenience sampling technique may lead to bias and jeopardize the generalizability of the outcomes. As a result, a stratified sampling could add to the study's representativeness. Self-reported data may introduce recall and reporting bias with socially favorable answers. So future studies can use more objective measures, such as smart watches and bracelets. Moreover, another limitation of this study was the lack of stratified analyses by age or gender, which limited the understanding of how the observed outcomes may differ across these demographic subgroups.

## Conclusions

This study revealed a high prevalence of physical inactivity among university students at KFU. A significant discrepancy was identified across academic disciplines, with students in medical programs demonstrating considerably lower levels of physical activity than their non-medical counterparts. Furthermore, the findings establish a robust link between lifestyle behaviors and activity levels, identifying smoking as a significant independent predictor of sedentarism. A future longitudinal study is warranted to establish casualty and reveal confounders. In conclusion, enrollment in a medical program and smoking status were the most critical factors associated with students' failure to meet the physical activity guidelines recommended by the WHO in this cohort.
